# The Role of XPG in Processing (CAG)_n_/(CTG)_n _DNA Hairpins

**DOI:** 10.1186/2045-3701-1-11

**Published:** 2011-03-09

**Authors:** Caixia Hou, Tianyi Zhang, Lei Tian, Jian Huang, Liya Gu, Guo-Min Li

**Affiliations:** 1 Graduate Center for Toxicology and Markey Cancer Center, University of Kentucky College of Medicine, Lexington, KY 40536, USA; 2 College of Life Sciences, Wuhan University, Wuhan, PR China

## Abstract

**Background:**

During DNA replication or repair, disease-associated (CAG)_n_/(CTG)_n _expansion can result from formation of hairpin structures in the repeat tract of the newly synthesized or nicked DNA strand. Recent studies identified a nick-directed (CAG)_n_/(CTG)_n _hairpin repair (HPR) system that removes (CAG)_n_/(CTG)_n _hairpins from human cells via endonucleolytic incisions. Because the process is highly similar to the mechanism by which XPG and XPF endonucleases remove bulky DNA lesions during nucleotide excision repair, we assessed the potential role of XPG in conducting (CAG)_n_/(CTG)_n _HPR.

**Results:**

To determine if the XPG endonuclease is involved in (CAG)_n_/(CTG)_n _hairpin removal, two XPG-deficient cell lines (GM16024 and AG08802) were examined for their ability to process (CAG)_n_/(CTG)_n _hairpins *in vitro*. We demonstrated that the GM16024 cell line processes all hairpin substrates as efficiently as HeLa cells, and that the AG08802 cell line is partially defective in HPR. Analysis of repair intermediates revealed that nuclear extracts from both XPG-deficient lines remove CAG/CTG hairpins via incisions, but the incision products are distinct from those generated in HeLa extracts. We also show that purified recombinant XPG protein greatly stimulates HPR in XPG-deficient extracts by promoting an incision 5' to the hairpin.

**Conclusions:**

Our results strongly suggest that 1) human cells possess multiple pathways to remove (CAG)_n_/(CTG)_n _hairpins located in newly synthesized (or nicked) DNA strand; and 2) XPG, although not essential for (CAG)_n_/(CTG)_n _hairpin removal, stimulates HPR by facilitating a 5' incision to the hairpin. This study reveals a novel role for XPG in genome-maintenance and implicates XPG in diseases caused by trinucleotide repeat expansion.

## Background

Expansion of trinucleotide repeats (TNRs) is responsible for certain familial neurological, neurodegenerative and neuromuscular disorders, such as CAG repeat expansion-caused Huntington's disease [1-3]. In these diseases, symptom severity is proportional to the extent of TNR expansions after the number of repeats reaches a critical threshold. However, the mechanisms involved in TNR expansions are not fully understood. Because DNA expansions require DNA synthesis, TNR expansions must be associated with DNA metabolism, including replication and/or repair [1-3]. Previous studies have suggested that TNR expansions could result from strand slippage-caused hairpin formations within TNR sequences (particularly CAG and CTG repeats) in the newly synthesized DNA strand during DNA replication or repair [1-7]. Indeed, CAG and CTG repeats can form very stable hairpin structures *in vitro *[8-10]; in addition, a recent elegant study by Liu et al. [11] provides evidence that the CAG/CTG hairpin can also occur *in vivo*, in a manner dependent on DNA replication. Therefore, timely removal of CAG/CTG hairpins during DNA metabolism is critical for maintaining TNR stability.

Recent studies have shown that human cells possess a repair system, referred to as DNA hairpin repair (HPR), that catalyzes error-free removal of CAG/CTG hairpins in a nick-dependent manner [12,13]. Interestingly, regardless of the strand location of the CAG/CTG hairpins, the HPR system always targets the nicked (i.e., newly synthesized) DNA strand for incisions, mainly using structure-specific endonucleases [13]. If the hairpin is located in the nicked strand, the repair system removes the hairpin either by making dual incisions flanking the heterology or by a combination of nick-directed excision and flap endonucleolytic cleavage, which leaves a small single-strand gap. If the hairpin is located in the continuous strand, incisions occur opposite the hairpin, followed by hairpin unwinding, which generates a relatively large single-strand gap. In either case, the gap is filled by replicative DNA polymerases using the continuous strand as a template [13]. As a result, the HPR system ensures TNR stability.

Use of dual incisions to remove CTG hairpins from the nicked strand [13] is highly similar to the manner in which the nucleotide excision repair (NER) pathway eliminates bulky DNA lesions [14,15]. NER is a very important cellular mechanism that prevents mutations by recognizing and removing the vast majority of bulky DNA adducts caused by ultraviolet irradiation and chemical agents. The NER reaction involves adduct recognition, adduct cleavage via dual incision, damaged fragment unwinding, and is completed by gap-filling DNA synthesis [14,15]. The dual-incision reaction is conducted by XPG and XPF-ERCC1, which are responsible for 3' and 5' cleavages, respectively [14,15]. While the dual incision mechanisms in NER and HPR are similar, it is not known if they are related.

In this study, we analyzed the HPR activity in two XPG-deficient cell lines derived from patients with xeroderma pigmentosum (XP) and/or Cockayne Syndrome. We show that human cells possess multiple dual incision mechanisms to remove CAG/CTG hairpins; and that while XPG is not essential for HPR, it stimulates CAG/CTG HPR by promoting hairpin incisions.

## Results

### XPG is not essential for (CAG)_25 _or (CTG)_25 _hairpin removal

**Figure 1:**
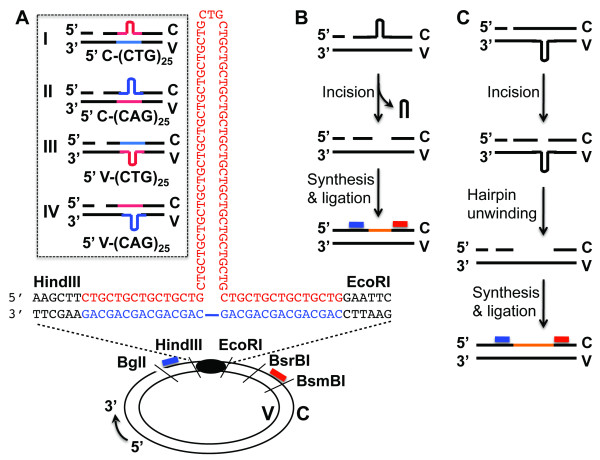
**DNA substrates and hairpin repair assays**. (A) DNA hairpin substrates. Circular DNA hairpin heteroduplexes were constructed using the M13mp18 bacterial phage series as described [13] (also see Methods for details). The blue and red types/lines represent CAG and CTG repeats, respectively, which are located between HindIII and EcoRI restriction enzyme sites of the plasmid. Each substrate contains a (CAG)_25 _or (CTG)_25 _hairpin either in the complementary (C) or viral (V) strand and a strand break (at the BglI recognition position) 5' to the hairpin. Substrates with a (CAG)_25 _or (CTG)_25 _hairpin in the V strand were named V-(CAG)_25 _or V-(CTG)_25_, respectively, while substrates with a (CAG)_25 _or (CTG)_25 _hairpin in the C strand were referred to as C-(CAG)_25 _or C-(CTG)_25_, respectively. Blue and red bars represent oligonucleotide probes that anneal to the nicked strand near the BglI and BsmBI sites, respectively. (B and C) Schematic diagrams of hairpin repair (HPR) assays. Given that CAG/CTG HPR only occurs in the nicked strand via incisions, followed by DNA resynthesis using the continuous strand as a template [12,13], the repair would result in either the hairpin sequence removal (B) or addition (C) depending on if the hairpin is located in C or V strand, respectively. The change in DNA length can be detected by Southern hybridization using an oligonucleotide probe (i.e., the blue or red bar) specifically annealing to the nicked strand.

Removal of (CTG)_n _hairpins via dual incision in HPR resembles the mechanism by which bulky DNA lesions are cleaved during nucleotide excision repair, where XPG is responsible for the 3' incision of a lesion. We therefore examined nuclear extracts of two XPG-deficient cell lines (AG08802 and GM16024) for their ability to process various (CAG)_25 _and (CTG)_25 _hairpin substrates (Figure 1). AG08802 is a lymphoblastoid cell line derived from an XP patient who inherited from his father an *XPG *gene coding for only the first 10 amino acid residues of the protein and obtained an *XPG *allele from his mother that transcribes little message [16]. GM16024 was transformed from lymphocytes of a female XP patient who carried a C to T transition in exon 4 in her paternal *XPG *allele that leads to a truncated XPG and a G to A transition in codon 874 in her maternal allele that converts alanine_874 _to threonine_874 _[17]. While AG08802 cells express no functional XPG, GM16024 cells retain an XPG activity capable of partially complementing the NER defect of XPG cells [17].

**Figure 2:**
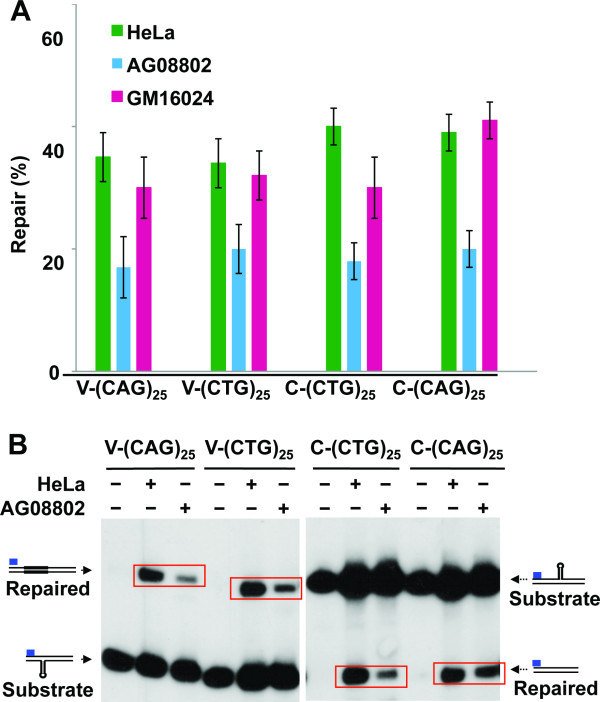
**HPR proficiency in cells defective in XPG**. (A) HPR efficiency in extracts derived from HeLa cells and two XPG-deficient cell lines, AG08802 and GM16024. The repair values were determined from at least three independent HPR assays. (B) AG08802 cells are partially defective in HPR. HPR assays were performed by incubating nuclear extracts derived from AG08802 or HeLa cells with individual hairpin substrates, as indicated. The resulting DNA products were digested with BglI and BsrBI and subjected to Southern blot analysis using a probe (blue bar) specifically annealing to the nicked strand near the BglI site. Schematic diagrams for the V- or C-strand hairpin repair are depicted in the left or right side of the gel, respectively, with repair products being highlighted by a red rectangle.

Because HPR is targeted to the nicked DNA strand, we scored HPR in this study by monitoring repeat-length changes in the nicked strand using a strand-specific ^32^P-labeled oligonucleotide probe as described [13]. Interestingly, the nuclear extract from GM16024 cells could process all hairpin substrates as efficiently as the HeLa nuclear extract (Figure 2A). By contrast, the extract from AG08802 cells possessed an HPR activity much weaker than that in HeLa nuclear extracts (Figure 2A and 2B). These results suggest that although XPG is not essential for DNA hairpin or loop repair [12,18], it plays a role in HPR.

### XPG stimulates HPR by promoting hairpin incisions

**Figure 3:**
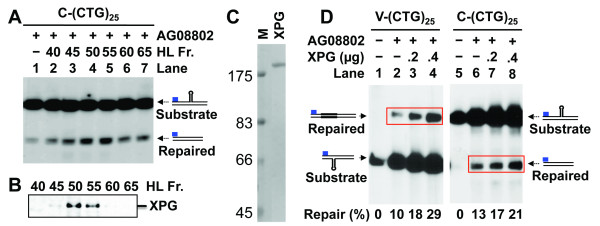
**Recombinant XPG protein stimulates HPR**. (A) A partially purified HeLa nuclear activity stimulates HPR by AG08802 extracts. Repair of substrate C-(CTG)_25 _was performed as described in the Figure 2 legend by mixing 75 μg of AG08802 nuclear extracts with 5 μl of individual HeLa fractions (HL Fr.) partially purified on a phosphocellulous P11 column [27]. (B) Western analysis of the HPR-stimulating P11 fractions with an antibody against XPG. (C) Coomassie staining of an SDS polyacrylamide gel showing purified recombinant XPG protein. (D) HPR assays on substrates V-(CTG)_25 _and C-(CTG)_25 _by AG08802 nuclear extracts in the presence or absence of purified XPG protein, as indicated. Repair products are highlighted by red rectangles.

To test the possibility that the weak HPR activity in AG08802 cells is due to loss of either XPG or a required HPR component, we examined partially purified HeLa nuclear fractions (chromatographed on a phosphocellulose column) for their ability to stimulate HPR in AG08802 extracts. We indeed identified an activity capable of stimulating AG08802 HPR (Figure 3A), but interestingly, the stimulating activity co-purified with XPG (Figure 3B). Thus, the purified recombinant XPG protein (Figure 3C) was examined for possible roles in stimulating CAG/CTG hairpin removal. As shown in Figure 3D, the HPR activity of AG08802 was greatly enhanced by addition of purified recombinant XPG protein; the stimulation is not only for substrate C-(CTG)_25 _(lanes 5-8), whose hairpin is removed via dual incisions in HeLa extracts [13], but also for substrate V-(CTG)_25 _(lanes 1-4), which undergoes a single incision in the non-hairpinned strand [13]. These observations support the idea that XPG either directly or indirectly participates in CAG/CTG hairpin removal.

**Figure 4:**
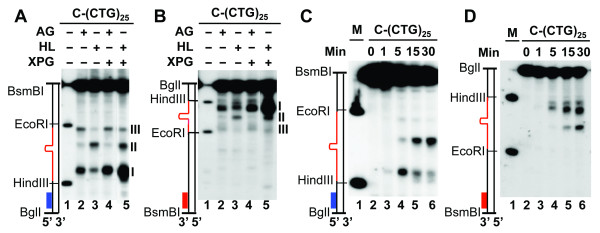
**XPG stimulates a 5' incision in C-(CTG)_25 _HPR**. (A) and (B) Analysis of HPR intermediates. HPR assays were performed using HeLa (HL) or AG08802 (AG) extracts under the condition of no DNA synthesis in the presence or absence of 0.3 μg of the purified recombinant XPG protein. The resulting DNA products were digested with BglI and BsmBI, followed by Southern blot analysis using an oligonucleotide probe specifically annealing to the nicked strand either near the BglI site (i.e., blue bar in A) or near the BsmBI site (i.e., red bar in B). (C and D) Time course analysis of incision intermediates of substrate C-(CTG)_25 _processed in HeLa extracts using the blue (C) or red (D) probe.

The mechanism by which XPG is involved in HPR was analyzed by monitoring HPR intermediates generated in AG08802 extracts in the absence or presence of purified XPG protein under conditions (e.g., no dNTPs) supporting DNA incision/excision, but not repair DNA synthesis as described [13,19]. Whereas we did not observe obvious differences in incision intermediates for substrate V-(CTG)_25 _between reactions with HeLa and AG08802 extracts (see below for detail), different incision patterns were identified when these extracts were incubated with substrate C-(CTG)_25_. As expected, HeLa extracts removed the C-(CTG)_25 _hairpin via dual incision, one 5' and the other 3' to the hairpin, generating products I and II, respectively (Figure 4A, lane 3, and [13]). Substrate C-(CTG)_25 _also underwent dual incisions in AG08802-containing reaction; however, the 3' incision occurred mainly in a manner that generated product III (Figure 4A, lane 2), instead of product II in HeLa extracts. These observations suggest that a (CTG)_n _hairpin located in the nicked strand can be removed by different dual incision mechanisms (see below for detail). Because XPG is known for its role in introducing a 3' nick to a bulky DNA lesion during NER [14,20], the simplest explanation for the discrepancy between reactions with HeLa and AG08802 extracts is that XPG is responsible for the 3' incision, which generates product II.

To explore this possibility, we compared HPR intermediates in AG08802 and HeLa extracts supplemented with purified recombinant XPG. To our surprise, XPG enhanced the production of the 5' incision (i.e., product I), but not the 3' incision (i.e., product II) in both the AG08802 and HeLa reactions (Figure 4A, lanes 4 and 5). To determine if the enhanced 5' incision is actually a subsequent event that requires an incision 3' to the hairpin by XPG (e.g., the cleavage that generates product II), the same reactions were performed, but the intermediates were detected using a ^32^P-labeled probe near the BsmBI site (see red bars in Figure 1). We did not detect a 3' incision stimulated by XPG; instead, the enhanced band is still product I, which is near the HindIII site (Figure 4B). In fact, our time-course experiments using HeLa extracts revealed that it is the 5' incision, but not the 3' incision, that occurs initially (Figure 4C and 4D), and this is consistent with previous observations [13]. We therefore conclude that XPG participates in CAG/CTG HPR by facilitating incisions that lead to hairpin removal.

### Multiple pathways for nick-strand hairpin removal

**Figure 5:**
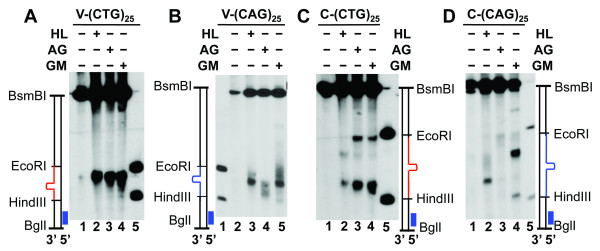
**Human cells possess multiple HPR pathways for nick strand hairpins**. HPR assays were performed in nuclear extracts from HeLa (HL), AG08802 (AG), or GM16024 (GM) under conditions of no DNA synthesis. The resulting DNA products were digested with BglI and BsmBI, and analyzed by Southern blot analysis using an oligonucleotide probe annealing to the nicked strand near the BglI site. The repair intermediates for substrates V-(CTG)_25_, V-(CAG)_25_, C-(CTG)_25_, and C-(CAG)_25 _are shown in panels A, B, C and D, respectively.

The distinct difference in incision patterns for substrate C-(CTG)_25 _between reactions with HeLa and AG08802 extracts raises the question of whether human cells possess multiple HPR pathways for each hairpin heteroduplex. To explore this question, we compared repair intermediates of individual hairpin substrates that were produced in HeLa extracts and extracts of two XPG-deficient cell lines (i.e., AG08802 and GM16024). The results show that consistent with the observations in HeLa cells, both XPG mutants removed all CAG/CTG hairpins via incisions (Figure 5). This is because a nick-directed excision would have destroyed the sequence to which the ^32^P-labeled probe anneals. Interestingly, all three extracts processed substrates V-(CTG)_25 _and V-(CAG)_25 _(two substrates containing a hairpin in the non-nicked strand) in a similar manner, since almost identical incision products for a given substrate were observed in reactions with individual extracts (Figure 5A and 5B). However, when these extracts were incubated with two substrates that contain a hairpin in the nicked strand (i.e., C-(CTG)_25 _and C-(CAG)_2_), different incision products were observed. Despite the two XPG mutants possessing different HPR activities (Figure 2A), they produced identical incision products for substrate C-(CTG)_25 _(Figure 5C, also see Figure 4A). For substrate C-(CAG)_25 _(Figure 5D), incision intermediates from all three reactions are different. The reaction containing HeLa extracts generated a major incision product as previously described [13] (also see Figure 5D, lane 2), the AG08802 reaction produced a product (slightly smeared) near the EcoRI site (Figure 5D, lane 3), and the substrate appeared to mainly undergo dual incisions in the reaction with GM16024 extracts (Figure 5D, lane 4). These results strongly suggest that human cells possess multiple pathways to remove a CAG/CTG hairpin located in the nicked (or newly synthesized) DNA strand.

## Discussion

In this study, we investigated the CAG/CTG HPR capacity of cells defective in XPG, which is one of the two endonucleases responsible for removing bulky DNA lesions via a dual incision mechanism [14,15]. Two interesting observations are made: 1) although XPG is not essential for CAG/CTG hairpin removal, it directly or indirectly participates in HPR by stimulating hairpin cleavage; and 2) human cells possess multiple incision pathways for removing CAG/CTG hairpins formed in the newly synthesized (or nicked) DNA strand.

Previous studies in HeLa nuclear extracts have revealed that CAG/CTG hairpins are mainly removed via endonucleolytic cleavages [13]. In this study, we show that incisions are also the primary mechanism by which XPG-deficient cells process CAG/CTG hairpins, which also supports the idea that NER enzymes are not essential for large loop or hairpin removal [12,18]. Analysis of HPR intermediates reveals that these XPG-deficient cells appear to remove CAG/CTG hairpins formed in the template (i.e., non-nicked) DNA strand in a manner similar to HeLa cells, since almost identical incision products were observed in reactions with HeLa, AG08802, and GM16024 extracts for substrates V-(CTG)_25 _and V-(CAG)_25 _(Figure 5A and 5B). However, the repair intermediates from two XPG mutants significantly differ from those in HeLa cells when processing hairpin structures formed in the nicked (or newly synthesized) DNA strand, i.e., substrates C-(CTG)_25 _and C-(CAG)_25 _(Figure 5C and 5D). Interestingly, although these XPG mutants process substrate C-(CTG)_25 _in an identical way (Figure 5C), they employed different mechanisms for C-(CAG)_25 _hairpin removal (Figure 5D), suggesting that human cells possess multiple pathways for removing CAG/CTG hairpins formed in the newly synthesized DNA strand. However, the enzymes involved in these alternative pathways and the mechanisms regulating the pathway choice remain to be investigated. Given the difference in cell types (epithelium for HeLa, lymphoblast for AG08802, and lymphocyte for GM16024), it is possible that these pathways are tissue/cell type-specific.

It is worth mentioning that despite a given cell extract showing a dominant HPR pathway for removing hairpins in the nicked strand, we did detect residual incision products of an alternative pathway in the same reaction -- e.g., residual product II in the AG08802-containing reaction and residual product III in the HeLa-containing reaction (Figure 4A). Based on the status of XPG in these cells, it is reasonable to believe that XPG is preferentially responsible for the processing in HeLa cells, but an alternative pathway takes place in the absence of XPG (e.g., in AG08802 and GM16024 cells). However, exogenous XPG failed to restore product II to reactions with XPG-deficient extracts (Figure 4A; data for GM16024 are not shown). Previous studies have revealed that these mutant cell lines express abnormal XPG proteins [16,17]. Thus, it is possible that these abnormal XPG proteins in AG08802 and GM16024 cells may have a dominant-negative role to inhibit the HPR pathway involving XPG by interacting with an XPG partner.

We also show that although XPG is not required for CAG/CTG hairpin removal, exogenous XPG significantly stimulates HPR by promoting an incision 5' to the hairpin (Figure 4). This is totally unexpected, because it is XPF, but not XPG, that conducts the 5' incision in NER [14,15]. How XPG, which makes the 3' incision in NER, promotes an incision 5' to the hairpin in HPR is unclear. Previous studies have shown that XPG stimulates base excision repair by facilitating the recruitment of DNA glycosylase/lyase to the damage site [21,22]. XPG was also shown to stabilize the TFIIH complex, thereby enhancing gene transcription [23]. The enzyme recruitment and stabilization activities associated with XPG could be responsible for its stimulation activity in HPR. Further studies are required to address these possibilities.

## Conclusions

Our research shows that human cells possess multiple pathways for CAG/CTG hairpin removal, especially for hairpins located in the newly synthesized strand. Although XPG is not essential for CAG/CTG hairpin removal, it stimulates HPR by facilitating a 5' incision to the hairpin. The work described here has revealed a novel role for XPG in genome-maintenance and implicated the enzyme in trinucleotide repeat expansion-caused diseases.

## Methods

### Cell culture and nuclear extract preparation

Cell lines HeLa S_3_, AG08802, and GM16024 were grown in RPMI 1640 medium supplemented with 10% fetal bovine serum (Hyclone) and 4 mM glutamine at 37° C in a 5% CO_2 _atmosphere to a density of 5 × 10^5 ^cells per ml. Nuclear extracts of each cell line were prepared as described [24].

### Preparation of (CAG)_n_/(CTG)_n _hairpin substrates

Circular heteroduplex substrates containing either a (CAG)_25 _or a (CTG)_25 _hairpin and a nick 5' to the hairpin in the complementary strand were prepared using bacterial phage series M13mp18-(CAG)_35_, M13mp18-(CTG)_10_, M13mp18-(CTG)_35_, M13mp18-(CAG)_10 _as described [13]. Substrates with a (CAG)_25 _or (CTG)_25 _hairpin in the viral strand were named V-(CAG)_25 _or V-(CTG)_25_, respectively, while substrates with a (CAG)_25 _or (CTG)_25 _hairpin in the complementary strand were referred to as C-(CAG)_25 _or C-(CTG)_25_, respectively (see Figure 1).

### Hairpin repair assay and analysis of repair intermediates

CAG/CTG hairpin repair was conducted essentially as described [13]. Briefly, 42 fmol of DNA heteroduplex were incubated with 100 μg of nuclear extracts in a 40-μl reaction containing 20 mM Tris-HCl (pH7.6), 110 mM KCl, 5 mM MgCl_2_, 1.5 mM ATP, 1 mM glutathione and 0.1 mM each of the four dNTPs at 37° C for 30 min. Reactions were terminated by adding protease K (30 μg/ml) and followed by sequential phenol extraction and ethanol precipitation. The recovered DNA was digested with BsrBI and BglI and fractionated through a denaturing polyacrylamide gel (6%), followed by electro-transferring to a nylon membrane. We probed the membrane with a ^32^P-end labeled oligonucleotide that annealed specifically to the BsrBI-BglI fragment in the nicked strand (see Figure 1) to score for conversion of 35 CAG/CTG repeats to 10 CAG/CTG repeats or vice versa. We visualized the repair products, as well as unrepaired molecules, by exposing the blots to X-Ray film. Repair efficiency was quantified by Kodak Molecular Imaging Software (version 5).

To investigate the incision intermediates, we conducted the *in vitro *repair assay as described above, but in the absence of exogenous dNTPs and in the presence of 0.15 mM aphidicolin. The recovered DNA samples were then digested, separated, and analyzed by Southern hybridization as described above.

In XPG complementation reactions, we used an XPG:extract ratio of 0.004:1 as previously described [25], i.e., for every 1.0 μg of nuclear extract, 4.0 ng of recombinant XPG was added.

### Purification of XPG protein and its activity assay

Human recombinant XPG was expressed in insect cells using the XPG baculoviral construct (provided by Drs. Joyce Reardon and Aziz Sancar, University of North Carolina) and purified essentially as described [26]. The activity of the purified XPG protein was assayed by virtue of its ability to cleave a bubble DNA substrate as described [26]. The purified protein is near homogeneity and displays a single polypeptide in an SDS PAGE stained with Coomassie Brilliant Blue (Figure 3C).

## Competing interests

The authors declare that they have no competing interests.

## Authors' contributions

CH carried out the majority of the experiments in this study and helped draft the manuscript. TZ participated in the XPG complementation experiments. LT participated in the expression and purification of XPG. JH participated in the experimental design and data analysis. LG designed and constructed the hairpin substrates, developed the *in vitro *HPR assay, and participated in writing the manuscript. GML conceived of the study, participated in the study design and data analysis, and wrote the manuscript. All authors read and approved the final manuscript.
